# Impact of Cranioplasty on Rehabilitation Course of Patients with Traumatic or Hemorrhagic Brain Injury

**DOI:** 10.3390/brainsci13010080

**Published:** 2022-12-31

**Authors:** Chiara Mele, Anna Bassetto, Valentina Boetto, Antonio Nardone, Valeria Pingue

**Affiliations:** 1 Department of Clinical-Surgical, Diagnostic and Pediatric Sciences, University of Pavia, 27100 Pavia, Italy; 2 Istituti Clinici Scientifici Maugeri IRCCS, Neurorehabilitation and Spinal Unit of Pavia Institute, 27100 Pavia, Italy; 3 Istituti Clinici Scientifici Maugeri IRCCS, Neurorehabilitation Unit of Montescano Institute, 27040 Montescano, Italy

**Keywords:** cranioplasty, hemorrhagic stroke, traumatic brain injury, rehabilitation, outcome

## Abstract

Background: Some authors have hypothesized that cranioplasty after decompressive craniectomy (DC) could positively influence functional recovery through several mechanisms. However, only a few studies with small sample sizes have investigated the effects of cranioplasty on functional recovery. Our study aims at evaluating the role of post-DC cranioplasty in influencing the functional recovery in a large cohort of patients with different etiologies of acquired brain injury (ABI). Methods: This retrospective study consecutively enrolled 253 patients with ABI, consisting of 108 adults who underwent post-DC cranioplasty and 145 adults who did not. All the subjects underwent a 6-month individual rehabilitation program. Demographic data, etiology, classification and anatomical site of brain injury, neurological and functional assessment at baseline and on discharge, and number of deaths during hospitalization were recorded. Results: In our cohort, 145 patients (57.3%) and 108 patients (42.7%) had, respectively, a hemorrhagic stroke (HS) and a traumatic brain injury (TBI). Only in the patients with TBI cranioplasty emerged as an independent predictor of better functional outcome in terms of the Functional Independence Measure (FIM) total score at discharge (β = 0.217, *p* = 0.001) and of the FIM variation during rehabilitation (ΔFIM) (β = 0.315, *p* = 0.001). Conversely, in the case of HS, no associations were found between post-DC cranioplasty and functional recovery. Conclusions: Post-DC cranioplasty was associated with better functional recovery six months after TBI but not in the patients with HS. Although the pathophysiological mechanisms underlying HS are different from those of TBI and possibly play a role in the different outcomes between the two groups, further studies are needed to investigate the mechanisms underlying the observed differences.

## 1. Introduction

Decompressive craniectomy (DC) represents a useful surgical procedure for reducing a high intracranial pressure (ICP) due to brain injury conditions, including traumatic brain injury (TBI) and subarachnoid or intracranial hemorrhages [1,2,3,4,5]. In addition to being effective in reducing uncontrolled intracranial pressure, DC is useful in improving brain oxygen delivery [6]. A large randomized controlled trial has also demonstrated a significant decrease in the mortality rate in patients with brain injury-related refractory high ICP treated with DC [7]. However, several studies have suggested that this surgical procedure could be associated with alterations in cerebrospinal fluid (CSF) circulation as well as with an impairment of glucose metabolism and cerebral blood flow [8,9]. Some authors hypothesized that the aforementioned alterations could negatively influence neurological and functional outcomes, although the clinical evidence remains controversial [10].

Cranioplasty after DC is a common neurosurgical procedure which contributes to the treatment or prevention of these complications and the restoration of the original skull contour [11,12,13]. The timing of cranioplasty can vary from weeks to months after craniectomy [4,5,6,7,8,9,10,11,12,13,14,15,16]. Generally, cranioplasty is performed 3–6 months post-DC, or longer in the case of infection of the surgical site [16]. Several studies demonstrated that cranioplasty normalizes CSF and regulates the perfusion dynamics by increasing cerebral blood flow in the major intracranial arteries, thus improving neurological deficits [12,17,18]. Moreover, cranioplasty increases the cerebrovascular reserve capacity in both hemispheres and the glucose metabolism of the injured hemisphere, as demonstrated using functional imaging [9,19,20]. In light of such evidence, some authors have hypothesized that cranioplasty could positively influence functional recovery [10,21,22]. However, only a few studies with small sample sizes have investigated the effects of cranioplasty on functional recovery, with many potential confounders that often complicate the interpretation of the results [10,21].

Therefore, the aim of our study was to evaluate the role of post-DC cranioplasty in influencing the functional recovery in a large cohort of patients with two different etiologies of acquired brain injury.

## 2. Materials and Methods

### 2.1. Study Design and Population

This observational retrospective cohort study enrolled 253 patients with traumatic brain injury (TBI) or hemorrhagic stroke (HS), consisting of 108 adults who underwent DC with subsequent cranioplasty (within 6 months of DC) and 145 adults who underwent DC without cranioplasty. All the subjects were admitted to the Neurorehabilitation Unit of Istituti Clinici Scientifici Maugeri IRCCS, Institute of Pavia, Italy, between 1 January 2009 and 31 December 2019.

The eligibility criteria included: (1) age  ≥  18 years; (2) diagnosis of TBI or hemorrhagic stroke (HS); (3) admission to a hospital emergency unit within 24 h after the traumatic event; (4) admission to our rehabilitation unit for an intensive neurorehabilitation program within one week from craniectomy to continue clinical care and rehabilitation programs started at the Acute Care Units of the Province of Pavia; and (5) continuation of inpatient neurorehabilitation program for at least 3 months after cranioplasty. Individuals were excluded from the study if the data concerning acute care were not available. We also excluded patients with pre-existing neurological events or diseases.

The selection criteria for cranioplasty included the occurrence of symptoms related to a craniectomy defect (i.e., syndrome of the trephined and/or seizures originating in the brain beneath the defect), the presence of pain or tenderness at the bone edges, and the need for cosmetic restoration of external skull appearance and symmetry, as well as the worsening of cognitive deficits.

The study design conformed to the ethical guidelines of the Declaration of Helsinki and was approved by the local Ethical Committee of Istituti Clinici Scientifici Maugeri (#2214 CE). The participants or authorized representatives signed a written informed consent form.

### 2.2. Variables, Data Sources, and Measurements

The data were retrieved from the electronic hospital records at the baseline and on discharge and included the following variables: sex, age at occurrence of brain injury, type of brain injury (TBI or HS), classification of lesion (cerebral edema or intracerebral hemorrhage, ICH, and/or subarachnoid hemorrhage, SAH), site of injury, associated neurosurgical procedures, neurological and functional assessment, and death during rehabilitation.

All the participants underwent an inpatient neurorehabilitation program consisting of individual 3 h daily treatment sessions, 6 days per week; the program included physiotherapy, occupational therapy, speech therapy, cognitive training, and nutrition assistance, as well as psychological and social support.

The Glasgow Coma Scale (GCS) and the Functional Independence Measure (FIM) scale were administered on admission (T0) and at discharge (T1) to evaluate the neurological and rehabilitation outcomes, respectively. The GCS represents a standardized tool for assessing the degree of neurological impairment and to define the seriousness of injury in relation to outcome, which involves three determinants: eye opening, verbal responses, and motor response or movement. These determinants are evaluated separately according to a numerical value which indicates the level of consciousness and the degree of dysfunction. The total scores range from 15 to 3. Patients are considered to have experienced a “mild” brain injury when their score is from 13 to 15. A score from 9 to 12 indicates a “moderate” brain injury, and a score equal to 8 or less reflects a “severe” brain injury [23].

The rehabilitation outcomes were evaluated through the FIM, an 18-item measurement scale that investigates the individual’s physical, psychological, and social functions [24,25]. This tool is useful for assessing the level of disability and any change in patient status in response to a rehabilitation program or medical intervention [26].

### 2.3. Statistical Analysis

The values are expressed as median and interquartile range (IQR) or absolute number and percentage. The data were tested for normality of distribution with the Shapiro–Wilk test and log-transformed when needed in order to correct for skewness. The Mann–Whitney U and chi-square tests were used for comparisons between the groups. Multiple linear regression analysis was used to evaluate the predictive role of cranioplasty on the rehabilitation outcome. The multilinear model included FIM T1 or ΔFIM as dependent variables and cranioplasty, sex, age, and brain injury characteristics as independent variables. The β coefficients and significant values obtained from the models were reported. Multivariate logistic regression analysis was used to identify the independent predictors of mortality during rehabilitation. The odds ratio (OR), 95% confidence interval (95% CI), and related significant values obtained from regression were reported. A value of *p*  <  0.05 was considered as statistically significant. Statistical analyses were performed using SPSS version 21 (IBM Corporation, Somers, NY, USA).

## 3. Results

### 3.1. Clinical and Functional Characteristics

A summary of the clinical and functional characteristics of the whole population is reported in Table 1. The male-to-female ratio was 1.5:1. Approximately, half of the patients were under 65 years of age at the time of acute brain injury. Overall, a hemorrhagic lesion was detected in 145 patients (57.3%), whereas a TBI was detected in 108 patients (42.7%). As a consequence of the hemorrhagic or traumatic lesion, the most frequent finding was SAH (34.0%), followed by ICH (32.8%), cerebral edema (27.3%), and ICH + SAH (5.9%). Regarding the localization of the injury, most of the patients (56.9%) presented multiple site lesions, with deep brain structures (20.1%) and frontal lobes (11.9%) being the most involved. Regarding neurosurgical interventions, 42.7% underwent cranioplasty within 6 months of craniectomy. Death during rehabilitative hospitalization was documented in 41 patients (16.2%).

Comparison analyses were conducted between the patients who underwent cranioplasty and those who did not (Table 1). Cranioplasty was more frequently performed the in females than in the males (χ^2^ = 7.2, *p* = 0.007) and in the case of HS than in TBI (χ^2^ = 11.3, *p* = 0.0008). As expected, the patients with SAH underwent cranioplasty more frequently than the patients with other lesions (χ^2^ = 9.2, *p* = 0.002). Moreover, the patients with involvement of the deep brain structures underwent cranioplasty less frequently than the patients with involvement of other brain structures (χ^2^ = 6.1, *p* = 0.01). Regarding mortality within 6 months of brain injury, the patients who underwent cranioplasty had a lower mortality rate than their counterparts (χ^2^ = 5.0, *p* = 0.02). The two groups were comparable with regard to age at diagnosis and neurological and functional outcomes.

Subsequently, the population was sub-grouped according to the type of brain injury (HS or TBI), as reported in Table 2. In the case of HS, cranioplasty was more frequently performed in the patients with SAH (χ^2^ = 19.8, *p* < 0.0001) and multiple site lesions (χ^2^ = 14.6, *p* < 0.0001), whereas it was performed less often in the case of ICH (χ^2^ = 25.2, *p* < 0.0001) and the involvement of deep brain structures (χ^2^ = 14.6, *p* = 0.0001) than in their counterparts. No significant differences were found in terms of sex, age, neurological and functional outcomes, and mortality within 6 months of injury.

**Figure 1:**
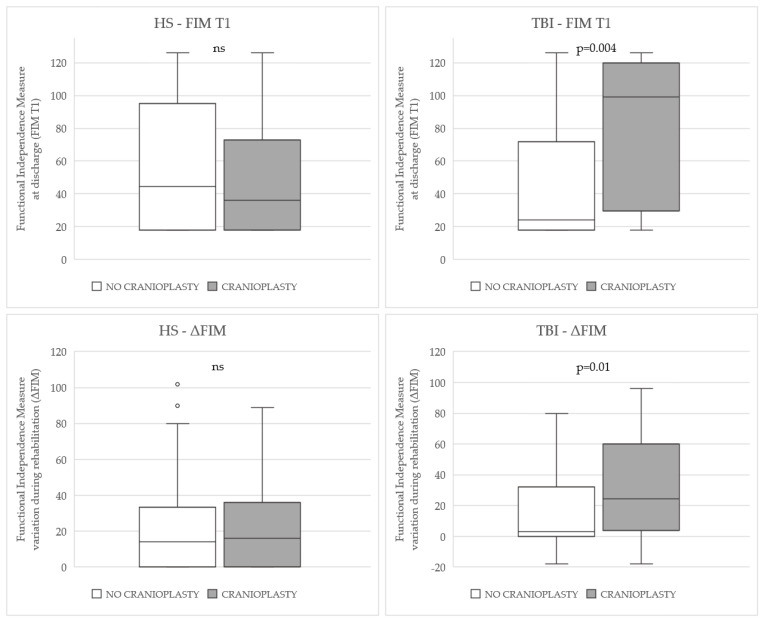
Tukey’s box-and-whisker plot of FIM T1 (upper boxes) and ΔFIM (lower boxes) in patients who underwent cranioplasty and those who did not, according to the type of brain injury (HS or TBI). Outliers are plotted as individual points. Abbreviation: ns, non-significant.

In the case of TBI, cranioplasty was more frequently performed in the patients ≤65 years of age than in the patients >65 years of age (χ^2^ = 3.8, *p* = 0.04). No significant differences were found in sex, type and site of lesion, and mortality within 6 months of injury. However, the patients who underwent cranioplasty had a higher improvement in functional outcome than their counterparts (FIM T1: *p* = 0.004, ΔFIM: *p* = 0.01) (Figure 1).

### 3.2. Post-DC Cranioplasty and Functional Outcome

Multiple linear regression analysis was conducted in the two subgroups of brain injury to evaluate the predictive role of cranioplasty on functional outcome. The models achieving the highest coefficient of determination (R^2^) are reported in Table 3.

Cranioplasty emerged as an independent predictor of a better functional outcome in terms of the FIM total score at discharge (FIM T1) and the FIM variation during rehabilitation (ΔFIM) only in the subgroup of patients with TBI. As expected, in both subgroups, the GCS severity at T0 and the FIM total score at T0 emerged as the main predictors of functional outcome. In HS, a better functional outcome was also independently predicted by male sex and a lower age at diagnosis.

### 3.3. Post-DC Cranioplasty and Mortality

Multivariable logistic regression analysis was conducted to evaluate the potential role of cranioplasty in influencing mortality during rehabilitation (Table 4). No significant association was found between cranioplasty and a reduced mortality rate in both subgroups. In TBI, the age at diagnosis emerged as the only predictor of mortality as it increased the risk of death fivefold (OR = 4.926, CI 95% 1.568–15.470, *p* = 0.006) during rehabilitation, whereas in the HS subgroup no associations were observed between mortality and the covariates considered in the analysis.

## 4. Discussion

The present study investigated the potential role of post-DC cranioplasty in influencing the functional recovery in a large cohort of patients with different etiologies of acquired brain injury. Our results show that post-DC cranioplasty represents an independent predictor of better functional outcome in patients with TBI. Conversely, in the case of HS, no associations were found between post-DC cranioplasty and functional recovery.

Although there is a controversial debate on the beneficial effects of post-DC cranioplasty, some authors have suggested a clinical and functional improvement in these patients [10,21,22]. However, only a few studies with small sample sizes investigated the effects of cranioplasty on functional recovery [10,21]. Our study confirmed the beneficial effects of cranioplasty in a large cohort of patients with TBI. TBI is sustained by heterogeneous pathophysiological mechanisms, which act synergically to contribute to the impairment of neurological and functional outcomes [27,28]. The primary damage promotes a cascade of metabolic, biochemical, and inflammatory alterations, leading to secondary injury, which is associated with vascular dysfunction, glutamatergic excitotoxicity, calcium overload, and neuroinflammation [28]. In this context, DC is effective in reducing cerebral edema and ICP, but at the same time, it could worsen post-TBI-related alterations.

It has been hypothesized that post-DC cranioplasty could improve neurological and functional outcomes through mechanisms that remain to be explored. Some authors have suggested that atmospheric pressure acts on the site of the cranial defect, altering cerebral activity [29]. The syndrome of the trephined represents a poorly understood complication of craniectomy, characterized by unexplained neurological dysfunction in patients with acquired skull defects [30]. Neurological deficits usually begin within weeks to months after craniectomy, and their occurrence is independent of the location of the primary lesion [30,31]. In this setting, post-DC cranioplasty could improve cerebral functions through a variety of mechanisms, including intracranial compliance restoration [32], ICP balancing [33,34], CSF hydrodynamic regulation [9], cerebral flow increasing [35,36], and metabolic changes [9]. The post-DC cranioplasty increase in global intracranial compliance, CSF circulation, and flow in the craniospinal junction promotes a more effective blood flow circulation and metabolism in the cerebral cortex, thus improving the motor and cognitive functions in these patients [22,37,38]. Moreover, this neurosurgical procedure is able to directly increase cerebral blood flow in the major intracranial arteries in both hemispheres, and as a result, a significant improvement in daily living activities and language functions was observed [19,20].

In a clinical study, Honeybul et al. observed a post-cranioplasty improvement in the FIM total score in only 4 of the 25 patients with TBI (16%) [39], whereas in our study more than 70% of the TBI subjects had an improvement in functional outcome. Our results partially agree with those of Jasey and co-workers, who demonstrated an improved function in about 60% of the patients [10]. The discrepancy between the findings is likely due to the limited sample size of previous studies and the heterogeneity of the outcome measures used. For this reason, some authors have pinpointed the need for a defined core outcome set (COS) to standardize the reporting in the research studies [40]. In addition, the previous studies often did not specify the rehabilitation program, and this contributes to the further difficulty in making comparisons with these studies. Finally, the lack of adjustment for important covariates in the previous studies limits the strength of the association between cranioplasty and functional recovery. In our regression model, we included several covariates in order to better define the role of cranioplasty in predicting functional outcome. Regardless of sex, age, classification and site of lesion, and GCS and FIM on admission, cranioplasty has emerged as an independent predictor of recovery.

These results were not confirmed in the subgroup of patients with HS. To date, the few studies that investigated the role of cranioplasty in influencing rehabilitation included only patients with trauma or small cohorts with different etiologies, without investigating the individual subgroups [41]. This is the first study that evaluates this issue in a large cohort of patients with HS. In this cohort, regardless of the potential confounders mentioned above, no associations were found between post-DC cranioplasty and functional recovery.

It is known that the pathophysiological mechanisms underlying HS are intrinsically different from those of TBI. In the former case, the damage is mainly related to the compressive effect of the hemorrhage on the cerebral parenchyma and to the effect of oxidative stress, neuronal apoptosis, and thrombin formation [42]. Moreover, in this subgroup, the prevalence of patients over 65 years of age with multiple comorbidities is higher than in the patients with TBI. Despite the potential role of cranioplasty in influencing functional recovery, in clinical practice patients with HS generally have many potential individual and clinical factors that could influence rehabilitation, thus masking the effect of cranioplasty. Therefore, further clinical trials will be required to elucidate the potential role and effect of cranioplasty in the rehabilitation setting [43].

Our study has several limitations, which should be pointed out. First, the study design does not allow us to draw any conclusion about the mechanisms involved in the relationship between cranioplasty and functional recovery in TBI. Second, the retrospective nature implies the review of clinical records not originally aimed at collecting data for research, with a risk of selection and recall biases and missing information. For instance, the data on the individual risk factors and the Glasgow Outcome Scale-Extended (GOSE), which are useful for a better characterization of the clinical and rehabilitation outcomes, respectively, were unavailable. Third, we did not include the definition of brain injury severity according to the criteria proposed by the VA/DoD Clinical Practice Guideline for Management of Concussion/Mild Traumatic Brain Injury [44]: structural imaging (mild TBI: normal, moderate and severe TBI: normal or abnormal); loss of consciousness (mild TBI: 0–30 min, moderate TBI: >30 min and <24 h, severe TBI: >24 h); alteration of consciousness/mental state (mild TBI: a moment up to 24 h, moderate and severe TBI: >24 h); and post-traumatic amnesia (mild TBI: 0–1 day, moderate TBI: >1 and <7 days, severe TBI: >7 days). These criteria are born from the evidence showing that the pathophysiology, clinical history, and prognosis for mild TBI are different than those for moderate-to-severe TBI. Fourth, we did not evaluate the effect of the cranioplasty timing on neurological and functional recovery, even if the most recent meta-analysis concluded that cranioplasty is associated with significant functional improvements, regardless of timing [45]. Finally, we did not assess the health-related quality of life (i.e., the HRQoL/QOLIBRI instrument), which represents an important outcome variable in this setting [46,47].

Despite the stated limitations, our results contribute to the evidence showing that cranioplasty represents a potential factor in influencing the functional recovery in some categories of patients.

## 5. Conclusions

In conclusion, post-DC cranioplasty was associated with better functional recovery six months after TBI but not in the patients with HS. Although the pathophysiological mechanisms underlying HS are different from those of TBI and possibly play a role in the different outcomes between the two patient groups, further studies are needed to investigate the mechanisms underlying the observed differences.

## Figures and Tables

**Table 1:** Clinical and rehabilitation characteristics of the population as a whole and subdivided into two groups based on whether or not the cranioplasty surgery was performed.

Variables		Whole Population n = 253 n (%)	Cranioplasty		*p*-Value
			No n (%) 145 (57.3)	Yes n (%) 108 (42.7)	
Sex	M	153 (60.5)	**98 (67.6)**	**55 (50.9)**	**0.007**
	F	100 (39.5)	**47 (32.4)**	**53 (49.1)**	
Age	≤65	131 (51.8)	70 (48.3)	47 (43.5)	0.45
	>65	122 (48.2)	75 (51.7)	61 (56.5)	
Type of lesion	HS	145 (57.3)	**70 (48.3)**	**75 (69.4)**	**0.0008**
	TBI	108 (42.7)	**75 (51.7)**	**33 (30.6)**	
Classification of lesion	Cerebral edema	69 (27.3)	**47 (32.4)**	**22 (20.4)**	**0.03**
	ICH	83 (32.8)	**55 (37.9)**	**28 (25.9)**	**0.04**
	SAH	86 (34.0)	**38 (26.2)**	**48 (44.4)**	**0.002**
	ICH + SAH	15 (5.9)	5 (3.5)	10 (9.3)	0.05
Site of lesion	Frontal	30 (11.9)	17 (11.7)	13 (12.0)	0.94
	Temporal	20 (7.9)	8 (5.5)	12 (11.1)	0.10
	Parietal	4 (1.6)	3 (2.1)	1 (0.9)	0.47
	Occipital	4 (1.6)	3 (2.1)	1 (0.9)	0.47
	Deep brain structures	51 (20.1)	**37 (25.5)**	**14 (13.0)**	**0.01**
	Multiple sites	144 (56.9)	77 (53.1)	67 (62.1)	0.16
GCS T0	Mild	51 (20.2)	32 (22.1)	19 (17.6)	0.38
	Moderate	103 (40.7)	55 (37.9)	48 (44.4)	0.30
	Severe	99 (39.1)	58 (40.0)	41 (38.9)	0.74
GCS T1 Available for 212 patients	Mild	115 (54.2)	61 (53.0)	54 (55.7)	0.70
	Moderate	50 (23.6)	29 (25.2)	21 (21.6)	0.54
	Severe	47 (22.2)	25 (21.8)	22 (22.7)	0.87
FIM T0 [median (IQR)]		18 (18–29)	18 (18–25)	18 (18–32)	0.67
FIM T1 [median (IQR)]		37 (18–84)	34 (18–83)	44 (18–87)	0.20
ΔFIM [median (IQR)]		13 (0–36.3)	8 (0–34)	18 (0–39)	0.09
Death		41 (16.2)	**30 (20.7)**	**11 (10.2)**	**0.02**

Data are expressed as median and interquartile range (IQR) or absolute number and percentage. Comparisons between groups were performed with χ^2^ or Mann–Whitney U tests. Significant differences are shown in bold characters. Abbreviations: HS, hemorrhagic stroke; TBI, traumatic brain injury; ICH, intracerebral hemorrhage; SAH, subarachnoid hemorrhage; GCS, Glasgow Coma Scale; FIM, Functional Independence Measure; T0, on admission; T1, at discharge; IQR, interquartile range.

**Table 2:** Clinical and rehabilitation characteristics of the population sub-grouped according to the type of brain injury.

Variables		HS 145 Patients			TBI 108 Patients		
		No Cranioplasty 70 Patients	Cranioplasty 75 Patients	*p*-Value	No Cranioplasty 75 Patients	Cranioplasty 33 Patients	*p*-Value
Sex	M	42 (60.0)	33 (44.0)	0.06	56 (74.7)	22 (66.7)	0.39
	F	28 (40.0)	42 (56.0)		19 (25.3)	11 (33.3)	
Age	≤65	33 (47.1)	38 (50.7)	0.67	**37 (49.3)**	**23 (69.7)**	**0.04**
	>65	37 (52.9)	37 (49.3)		**38 (50.7)**	**10 (30.3)**	
Classification of lesion	Cerebral edema	0 (0.0)	0 (0.0)	-	48 (64.0)	21 (63.6)	0.11
	ICH	**55 (78.6)**	**28 (37.3)**	**<0.0001**	0 (0.0)	0 (0.0)	-
	SAH	**11 (15.7)**	**38 (50.7)**	**<0.0001**	26 (34.7)	11 (33.3)	0.89
	ICH + SAH	4 (5.7)	9 (12.0)	0.18	1 (1.3)	1 (3.0)	0.55
Site of lesion	Frontal	9 (12.9)	12 (16.0)	0.59	8 (10.7)	1 (3.0)	0.18
	Temporal	4 (5.8)	9 (12.0)	0.18	4 (5.4)	3 (9.1)	0.46
	Parietal	2 (2.8)	1 (1.3)	0.52	1 (1.3)	0 (0.0)	0.50
	Occipital	2 (2.8)	0 (0.0)	0.14	1 (1.3)	1 (3.0)	0.55
	Deep brain structures	**25 (35.7)**	**7 (9.4)**	**0.0001**	12 (16.0)	7 (21.2)	0.51
	Multiple sites	**28 (40.0)**	**46 (61.3)**	**0.01**	49 (65.3)	21 (63.7)	0.86
GCS T0	Mild	18 (25.7)	11 (14.7)	0.10	14 (18.7)	8 (24.3)	0.51
	Moderate	27 (38.6)	34 (45.3)	0.41	28 (37.3)	14 (42.4)	0.62
	Severe	25 (35.7)	30 (40.0)	0.59	33 (44.0)	11 (33.3)	0.30
GCS T1	Mild	34 (56.6)	36 (52.2)	0.61	27 (49.1)	18 (64.3)	0.19
	Moderate	13 (21.7)	17 (24.6)	0.69	16 (29.1)	4 (14.3)	0.13
	Severe	13 (21.7)	16 (23.2)	0.84	12 (21.8)	6 (21.4)	0.97
FIM T0 [median (IQR)]		18 (18–30)	18 (18–25)	0.20	**18 (18–20)**	**21 (18–49)**	**0.01**
FIM T1 [median (IQR)]		45 (18–95)	36 (18–74)	0.55	**24 (18–72)**	**99 (23–120)**	**0.004**
Δ FIM [median (IQR)]		14 (0–34)	16 (0–37)	0.94	**3 (0–35)**	**25 (1–60)**	**0.01**
Death		10 (14.3)	6 (8.0)	0.23	20 (26.7)	5 (15.2)	0.19

Data are expressed as median and interquartile range (IQR) or absolute number and percentage. Comparisons between groups were performed with χ^2^ or Mann–Whitney U tests. Significant differences are shown in bold characters. Abbreviations: HS, hemorrhagic stroke; TBI, traumatic brain injury; ICH, intracerebral hemorrhage; SAH, subarachnoid hemorrhage; GCS, Glasgow Coma Scale; FIM, Functional Independence Measure; T0, on admission; T1, at discharge; IQR, interquartile range.

**Table 3:** Multiple linear regression analysis to evaluate the potential association between cranioplasty and functional outcome in the population sub-grouped according to the type of brain injury.

			Dependent Variable FIMT1 Total Score		Dependent Variable ΔFIM Total Score	
HS	**Regression Models**		**Beta**	***p*-Value**	**Beta**	***p*-Value**
	Model 1 R^2^ FIMT1 = 0.52 R^2^ ΔFIM = 0.21	Sex	**−0.182**	**0.004**	**−0.197**	**0.02**
		GCS T0 severity	**−0.679**	**<0.0001**	**−0.395**	**<0.0001**
		Cranioplasty	−0.019	0.76	0.022	0.78
	Model 2 R^2^ FIMT1 = 0.51 R^2^ ΔFIM = 0.20	Age	**−0.135**	**0.03**	**−0.171**	**0.04**
		GCS T0 severity	**−0.695**	**<0.0001**	**−0.412**	**<0.0001**
		Cranioplasty	−0.040	0.52	0.011	0.90
	Model 3 R^2^ FIMT1 = 0.49 R^2^ ΔFIM = 0.17	Site of lesion	−0.053	0.42	0.015	0.86
		GCS T0 severity	**−0.688**	**<0.0001**	**−0.415**	**<0.0001**
		Cranioplasty	−0.035	0.59	−0.004	0.97
	Model 4 R^2^ FIMT1 = 0.50 R^2^ ΔFIM = 0.18	Classification of lesion	0.083	0.22	0.088	0.31
		GCS T0 severity	**−0.704**	**<0.0001**	**−0.421**	**<0.0001**
		Cranioplasty	−0.066	0.32	−0.028	0.74
	Model 5 R^2^ FIMT1 = 0.63 R^2^ ΔFIM = 0.22	GCS T0 severity	**−0.407**	**<0.0001**	**−0.590**	**<0.0001**
		FIM T0 total score	**0.473**	**<0.0001**	**−0.291**	**0.004**
		Cranioplasty	−0.013	0.81	−0.019	0.81
TBI	Model 1 R^2^ FIMT1 = 0.63 R^2^ ΔFIM = 0.31	Sex	0.067	0.33	0.099	0.29
		GCS T0 severity	**−0.711**	**<0.0001**	**−0.453**	**<0.0001**
		Cranioplasty	**0.278**	**<0.0001**	**0.277**	**0.004**
	Model 2 R^2^ FIMT1 = 0.64 R^2^ ΔFIM = 0.30	Age	−0.130	0.07	−0.059	0.54
		GCS T0 severity	**−0.706**	**<0.0001**	**−0.453**	**<0.0001**
		Cranioplasty	**0.248**	**0.001**	**0.263**	**0.008**
	Model 3 R^2^ FIMT1 = 0.62 R^2^ ΔFIM = 0.31	Site of lesion	0.029	0.68	0.092	0.34
		GCS T0 severity	**−0.711**	**<0.0001**	**−0.455**	**<0.0001**
		Cranioplasty	**0.283**	**<0.0001**	**0.291**	**0.003**
	Model 4 R^2^ FIMT1 = 0.62 R^2^ ΔFIM = 0.32	Classification of lesion	−0.025	0.73	−0.111	0.24
		GCS T0 severity	**−0.709**	**<0.0001**	**−0.450**	**<0.0001**
		Cranioplasty	**0.279**	**<0.0001**	**0.279**	**0.004**
	Model 5 R^2^ FIMT1 = 0.73 R^2^ ΔFIM = 0.35	FIM T0 total score	**0.444**	**<0.0001**	**−0.641**	**<0.0001**
		GCS T0 severity	**−0.422**	**<0.0001**	**−0.282**	**0.03**
		Cranioplasty	**0.217**	**0.001**	**0.315**	**0.001**

Sex: M = 0, F = 1; age: ≤65 = 0, >65 = 1; classification of lesion: ICH = 0, SAH = 1, ICH + SAH = 2, cerebral edema = 3; site of lesion: single lesion = 0, multiple lesion = 1; cranioplasty: no = 0, yes = 1; GCS T0 severity: mild = 0, moderate = 1, severe = 2. Significant associations are shown in bold characters. Abbreviations: HS, hemorrhagic stroke; TBI, traumatic brain injury; ICH, intracerebral hemorrhage; SAH, subarachnoid hemorrhage; GCS, Glasgow Coma Scale; FIM, Functional Independence Measure; T0, on admission; T1, at discharge.

**Table 4:** Multivariable logistic regression analysis showing the potential risk factors for mortality within 6 months of brain injury.

	Covariates	Death during Rehabilitation (Dependent Variable) (No = 0, Yes = 1)		
		OR	CI 95%	*p*-Value
HS	Sex	0.347	0.101–1.196	0.35
	Age	2.459	0.701–8.628	0.16
	Classification of lesion	1.072	0.391–2.936	0.89
	Site of lesion	0.188	0.021–1.724	0.14
	Cranioplasty	0.428	0.122–1.500	0.18
	GCS T0 severity	1.424	0.459–4.420	0.54
	FIM T0	0.754	0.478–1.188	0.22
TBI	Sex	0.474	0.132–1.708	0.25
	Age	**4.926**	**1.568–15.470**	**0.006**
	Classification of lesion	1.917	0.520–7.072	0.33
	Site of lesion	0.766	0.425–1.381	0.38
	Cranioplasty	1.005	0.275–3.671	0.99
	GCS T0 severity	1.279	0.467–3.502	0.63
	FIM T0	0.799	0.577–1.106	0.17

Sex: M = 0, F = 1; age: ≤65 = 0, >65 = 1; classification of lesion: ICH = 0, SAH = 1, ICH + SAH = 2, cerebral edema = 3; site of lesion: single lesion = 0, multiple lesion = 1; cranioplasty: no = 0, yes = 1; GCS T0 severity: mild = 0, moderate = 1, severe = 2. Significant associations are shown in bold characters. Abbreviations: HS, hemorrhagic stroke; TBI, traumatic brain injury; ICH, intracerebral hemorrhage; SAH, subarachnoid hemorrhage; GCS, Glasgow Coma Scale; FIM, Functional Independence Measure; T0, on admission.

## Data Availability

The data presented in this study are available on request from the corresponding author.
